# Variation Characteristics of the Main Hydrochemical Indexes in Typical Subterranean Rivers in the South China Karst Region Based on Curve Fitting

**DOI:** 10.1155/2019/2787349

**Published:** 2019-03-06

**Authors:** Shuyi Liu, Chuan Liang, Lina Wang, Lingwei Zeng, Chunyi Wang

**Affiliations:** ^1^College of Water Resource and Hydropower, Sichuan University, Chengdu, China; ^2^Department of Hydraulic Engineering, Guangdong Technical College of Water Resources and Electric Engineering, Guangzhou, China; ^3^College of Cyber Security, Sichuan University, Chengdu, China; ^4^The Engineering Department, Shanghai Foundation Engineering Group Co., Ltd, Shenzhen, China

## Abstract

The water quality of subterranean rivers in the South China Karst region has undergone dramatic changes resulting from industrial and social development over the past 60 years. The combination of sampling results from subterranean rivers in four typical study areas in the South China Karst region from October to December 2015 (dry season) and correlation analysis using SPSS revealed that the main ions K+, Na+, and SO42- exhibited a significant correlation (p<0.01) and that Ca2+, HCO3-, and Mg2+ exhibited a good correlation (p<0.01). Additionally, we consolidated the data collected since 1960 and, by applying MATLAB, a variety of fitting curve methods were used to fit all the data, and the results showed that cubic spline interpolation fitting performed the best. The squared correlation coefficients (R2) of the obtained fitting curves for Ca2+, HCO3-, and Mg2+ are 0.8545, 0.8689, and 0.7632, respectively, and the corrected R2 values are 0.6739, 0.7088, and 0.4853, respectively. The R2 values of the obtained fitting curves for K+, Na+, Cl-, SO42-, and NO3- are 0.9085, 0.8964, 0.7531, 0.6222, and 0.7997, respectively, and the corrected R2 values are 0.7904, 0.7669, 0.5272, 0.2815, and 0.6127, respectively, indicating excellent fits. Based on the fitting curves, the overall water quality conditions in the karst region were analyzed and compared. Finally, the development of subterranean rivers in the South China Karst region was determined. Based on the results, the following conclusions can be drawn: the development of the subterranean rivers is indeed a slow process, but water quality can change rapidly in response to the transformation of industrial society. Additionally, the results indicate the crucial importance of urban planning that takes into account environmental protection during development in karst regions. This study aims to provide a basis for the management of karst areas and the improvement of groundwater quality by evaluating geochemical processes.

## 1. Introduction

The water resources in the South China Karst region mainly exist as subterranean rivers, which have received increasing attention as water sources. Considering the hydrogeological conditions, widespread agricultural activities, improper treatment of industrial wastewater, and leakage of mining areas, a number of subterranean rivers are thought to be been contaminated [1–5]. In recent years, China has established urban planning that takes into account environmental protection, and it has issued various corresponding policies that contribute to resolving water contamination and improving overall water environments. The ultimate goal is to reach the water quality standards of developed countries. Therefore, it is imperative to investigate the development and changes of the main hydrochemical indexes under the impact of human activities.

In the past few decades, research on subterranean rivers in karst areas in South China has primarily included determining hydrogeological conditions using hydrochemical methods to trace pollution sources [6–10]. Moreover, most of the studies focused on a single river basin or subterranean river. Research that studies multiple subterranean rivers in neighboring river basins is rarely reported. Considering the unique hydrogeological conditions of the karst regions in South China, the distribution of subterranean rivers is unbalanced [11]. The karst regions in China can be divided into undeveloped, appropriately developed, and overdeveloped regions [12, 13]. Based on these three development states, the Jila subterranean river in Liuzhou, the Lihu subterranean river in Nandan, and the Maocun village subterranean river in Guilin in the karst regions of Guangxi Province and the Banzhai subterranean river in Maolan in the karst regions of Guizhou Province were chosen. In the present paper, the main hydrochemical indexes over many years were first fit. Based on the fitting curves, the overall water resource conditions in the karst regions were then analyzed and compared. Additionally, the development of subterranean rivers in the South China Karst region was determined. These results provide a scientific basis for the optimal management of subterranean river water resources and for the improvement of the water quality of subterranean rivers.

## 2. Overview of the Study Site

**Figure 1:**
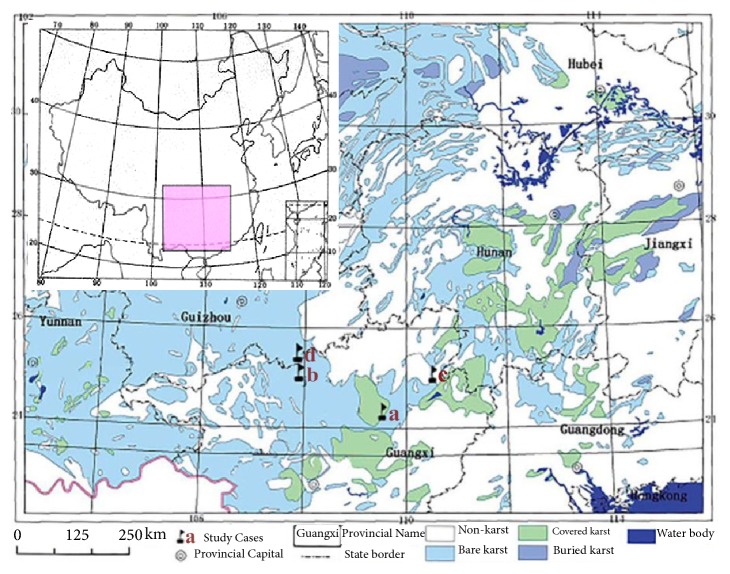
Location of the four studied rivers and the karst distribution in South China[10].

The locations of the Jila subterranean river in Liuzhou (a), Lihu subterranean river in Nandan (b), Maocun subterranean river in Guilin (c) in Guangxi Province, and the Banzhai subterranean river in Maolan (d) in Guizhou Province in South China are shown in Figure 1.

All four typical subterranean river basins in the South China Karst region feature the bare karst land cover type and are located in the same latitude zone of 21~26°N. Furthermore, these basins are located in the subtropical monsoon climate zone. The main source of groundwater recharge is atmospheric precipitation, which enters the karst groundwater system through sinkholes, karst windows, subterranean river inlets, and underground streams. Thus, the studied rivers are typical subterranean rivers in the South China Karst region but feature different land use patterns. The typical characteristics of the four subterranean rivers are as follows: the Jila subterranean river basin represents a city-dominated basin with industrial and domestic pollution, the Lihu subterranean river basin represents a town-dominated basin with mining and domestic pollution, the Maocun subterranean river basin represents a village-dominated basin with agricultural and domestic pollution, and the Banzhai subterranean river basin represents a nature reserve-dominated basin with domestic pollution.

During October-December 2015 (dry season), we conducted a field investigation of these four subterranean rivers and collected water samples. At present, these four subterranean river basins have been impacted by human activities to different degrees. The results from past studies and those from the current onsite investigation are described separately below.

### 2.1. Jila Subterranean River Basin in Liuzhou

The Jila subterranean river basin in Guangxi Province is located within the heavy industrial city of Liuzhou, which is experiencing rapid urban development. Zou et al. [14] performed a water resource assessment of the Jila subterranean river in 2006-2008 and established a long-term monitoring network for the water environment. The investigation revealed that there were a total of 28 notable pollution sources, including domestic sewage and industrial and agricultural pollution. The outlets of subterranean rivers have been used as the main drainage outlets for industrial water and domestic sewage in the river basin. At the time of the study, the Jila subterranean river basin had been severely polluted by industrial wastewater (including the heavy metals Mn and Fe) and domestic sewage (NH_4_^+^ and Cl^−^). Long et al. [15] also evaluated the subterranean rivers in this region during the same period. They found that the concentrations of Mn, SO_4_^2−^, and CN^−^ exceeded the water quality standard limits substantially and that the outlets of some subterranean rivers exhibited eutrophication.

**Figure 2:**
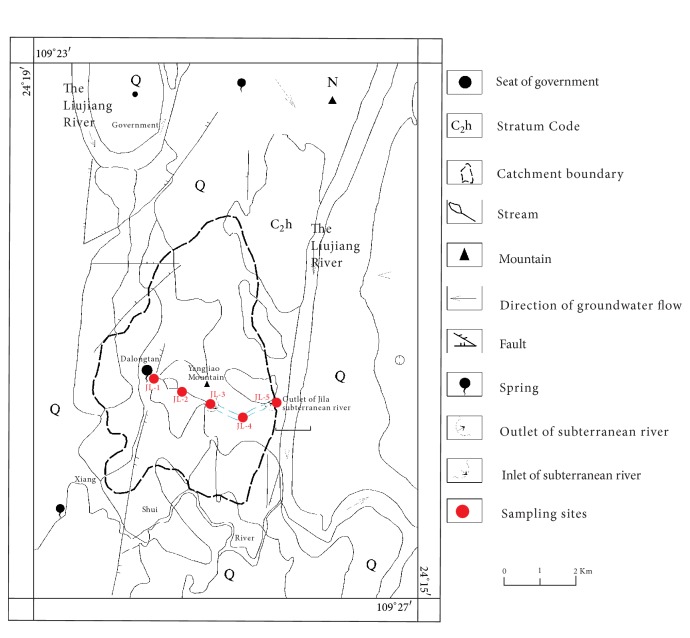
Hydrogeological map and location of sampling sites in the Jila subterranean river basin [15].

Currently, the city of Liuzhou is experiencing considerable construction and changes. The onsite investigation shows that all the obvious pollution point sources in the Jila subterranean river basin disappeared in 2008 and were replaced by underground sewage pipes in response to city planning. Five representative subterranean river outlets were chosen as study areas in the field investigation.  Figure 2 shows the hydrological-geological map and sampling locations for the Jila subterranean river basin in the present field investigation.

### 2.2. Lihu Subterranean River Basin in Nandan

The Lihu subterranean river basin in Nandan in Guangxi Province is an important water source that has multiple social and economic functions, including an urban water source, agricultural irrigation water source, and industrial water source [6]. The subterranean river in this river basin has many outlets.

**Figure 3:**
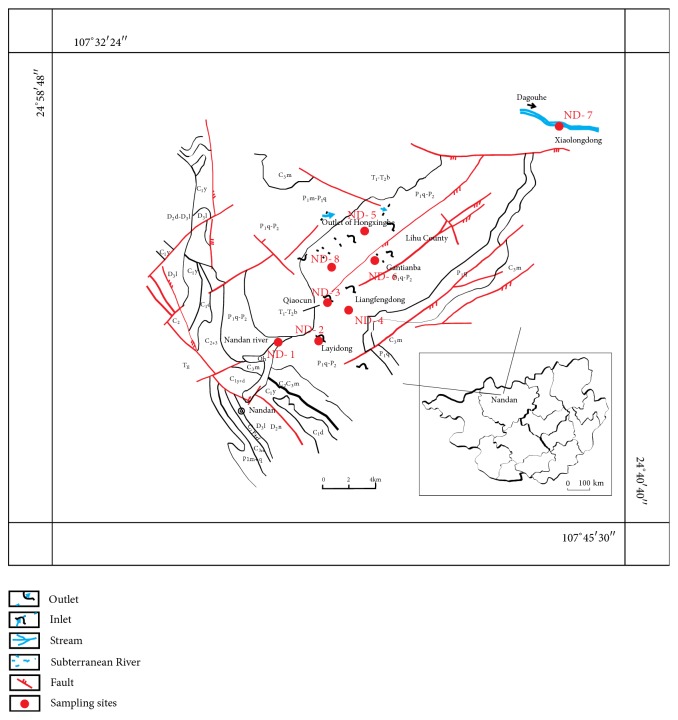
Hydrogeological map and location of sampling sites in the Lihu subterranean river basin [6].

Eight representative subterranean river outlets were chosen as study areas in the field investigation.  Figure 3 shows the hydrological-geological map and sampling locations in the Lihu subterranean river basin in Nandan.

### 2.3. Maocun Subterranean River Basin in Guilin

The Maocun subterranean river basin in Guangxi Province is a region with no heavy industry but with a concentrated population and agriculture as the main industry. As an experimental base for the Institute of Karst Geology, Chinese Academy of Geological Sciences (CAGS), many relevant studies have been conducted in the Maocun village subterranean river basin in Guilin, including the determination of the hydrogeological conditions using the tracing test technique [8] and the analysis of the carbon sink associated with karst[9].

**Figure 4:**
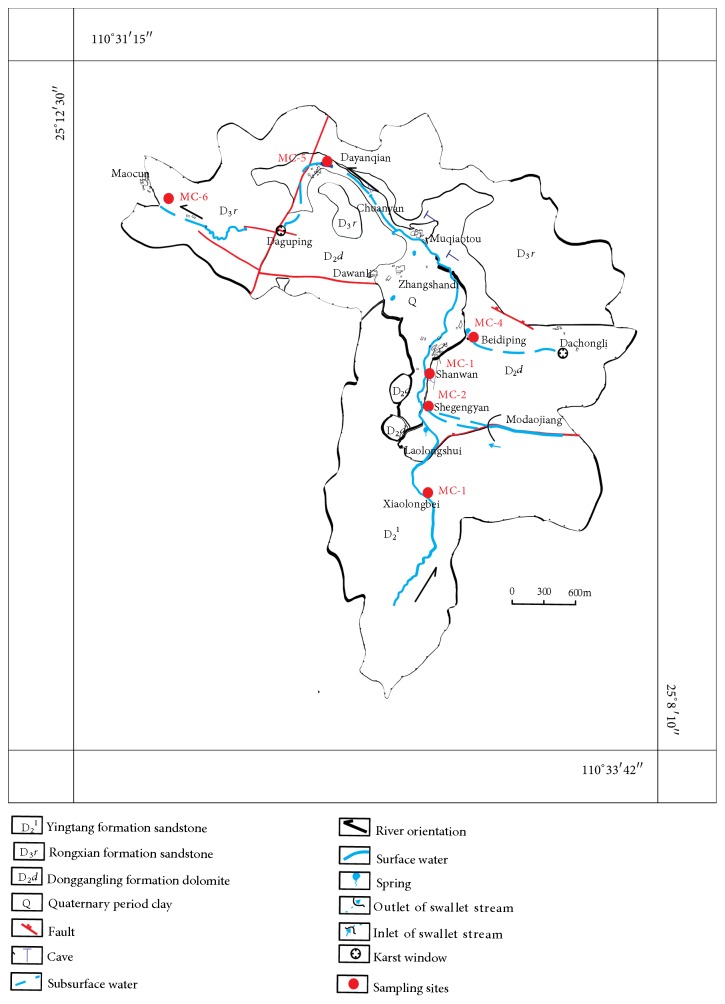
Hydrogeological map and location of sampling sites in the Maocun subterranean river basin.

Six representative subterranean river outlets were chosen as study areas in the field investigation.  Figure 4 shows the hydrological-geological map and sampling locations in the Maocun village subterranean river basin in Guilin.

### 2.4. Banzhai Subterranean River Basin in Maolan

The Banzhai subterranean river basin in Maolan in a karst region in Guizhou Province features a low level of development, and the population is dispersed.

**Figure 5:**
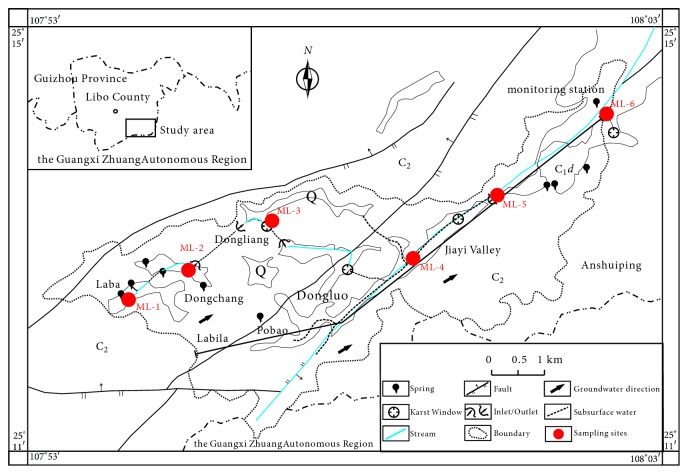
Hydrogeological map and location of sampling sites in the Banzhai subterranean river basin.

Six representative subterranean river outlets were chosen as study areas in the field investigation.  Figure 5 shows the hydrological-geological map and sampling locations in the Banzhai subterranean river basin in Maolan in Guizhou Province.

## 3. Water Sample Collection and Measurement

Water samples were collected from multiple sites in each subterranean river basin, including karst windows, sinkholes and outlets. A total of 25 samples (including samples from all sampling locations) were collected and tested to determine the hydrochemical indexes.

A multiparameter water quality analyzer (WTW350i, Germany) was used in the field to measure water temperature, dissolved oxygen (DO), pH, and electric conductivity (Ec) at resolutions of 0.1°C, 0.01 mg/l, 0.01 pH units and 1 *μ*S/cm, respectively. In addition, an alkalimeter and a hydrometer (Merck, Germany), with precisions of 0.1 mmol/L and 2 mg/L, were used to measure the HCO_3_^−^ and Ca^2+^ concentrations in water in the field.

The water samples were stored and sealed in polyethylene bottles and brought back to the laboratory for determination of the ion concentrations. All samples were stored in 600 mL polypropylene bottles before being shipped to the laboratory. Each bottle was sealed and stored below 4°C during the analysis. The measurement and analysis results for 8 major dissolved ions, including major cations (K^+^, Na^+^, Ca_2_^+^, and Mg_2_^+^) and major anions (HCO_3_^−^, Cl^−^, SO_4_^2−^, and NO_3_^−^), from the laboratory were certified by relevant governing bodies.

## 4. Water Sample Measurement Results

### 4.1. Jila Subterranean River Basin in Liuzhou (JL-1~JL-5)

The average pH and Ec for the 5 outlets in the river basin were 7.28 and 555.6 *μ*s/cm, respectively. The average concentrations of the main ions K^+^, Na^+^, Ca^2+^, Mg^2+^, HCO_3_^−^, Cl^−^, SO_4_^2−^, and NO_3_^−^ were 3.32 mg.L^−1^, 10.5 mg.L^−1^, 86.8 mg.L^−1^, 15.5 mg.L^−1^, 282.5 mg.L^−1^, 13.22 mg.L^−1^, 48.72 mg.L^−1^, and 14.03 mg.L^−1^, respectively.

### 4.2. Lihu Subterranean River Basin in Nandan (ND-1~ND-8)

The average pH and Ec for the 8 outlets in the river basin were 8.05 and 353.25 *μ*s/cm, respectively. The average concentrations of the main ions K^+^, Na^+^, Ca^2+^, Mg^2+^, HCO_3_^−^, Cl^−^, SO_4_^2−^, and NO_3_^−^ were 1.70 mg.L^−1^, 4.57 mg.L^−1^, 69.38 mg.L^−1^, 3.75 mg.L^−1^, 169.66 mg.L^−1^, 4.07 mg.L^−1^, 36.93 mg.L^−1^, and 9.11 mg.L^−1^, respectively.

### 4.3. Maocun Village Subterranean River Basin in Guilin (MC-1~MC-6)

Among the 6 outlets in this river basin, there were allogeneic water resources entering the karst region at sample locations MC-1~MC-3[17]. As a result, the concentrations of the main ions were lower than those for the other 3 sample locations. After excluding MC-1~MC-3, the average pH and Ec of the other 3 subterranean water sample locations were 7.24 and 391.67 *μ*s/cm, respectively. The average concentrations of the main ions K^+^, Na^+^, Ca^2+^, Mg^2+^, HCO_3_^−^, Cl^−^, SO_4_^2−^, and NO_3_^−^  were 0.46 mg.L^−1^, 0.43 mg.L^−1^, 85.67 mg.L^−1^, 10.66 mg.L^−1^, 279.17 mg.L^−1^, 1.32 mg.L^−1^, 5.98 mg.L^−1^, and 3.83 mg.L^−1^, respectively.

### 4.4. Banzhai Subterranean River Basin in Maolan in the Karst Regions of Guizhou Province (ML-1~ ML-6)

The average pH and Ec for the 7 outlets in the river basin were 7.7 and 397.83 *μ*s/cm, respectively. The average concentrations of the main ions K^+^, Na^+^, Ca^2+^, Mg^2+^, HCO_3_^−^, Cl^−^, SO_4_^2−^, and NO_3_^−^ were 0.79 mg.L^−1^, 0.44 mg.L^−1^, 63 mg.L^−1^, 18.98 mg.L^−1^, 261.46 mg.L^−1^, 1.51 mg.L^−1^, 7.90 mg.L^−1^, and 5.39 mg.L^−1^, respectively.

The measured concentrations for the main ions at the 25 sampling points of the 4 typical subterranean rivers are listed in Table 1.

## 5. Analysis and Discussion

### 5.1. Dilution Effect by Precipitation

Among the measurement results for the water samples collected from the four subterranean river basins, the measurements for all indexes reported as part of the study were lower than those reported for the same time period during previous years [6, 9, 14–16]. The results are listed in Tables 2~5. Based on the report of the annual precipitation in Guangxi and Guizhou Province between 1961 and 2015, the precipitation in the dry season for the four typical subterranean rivers in 2015 was the second highest in the past 60 years [18]. Therefore, the low measurement values for this study could be mainly attributed to the dilution effect associated with precipitation entering the subterranean rivers, which is in agreement with conclusions presented in the literature [10].

### 5.2. Hydrochemical Types in the Four Study Areas

**Figure 6:**
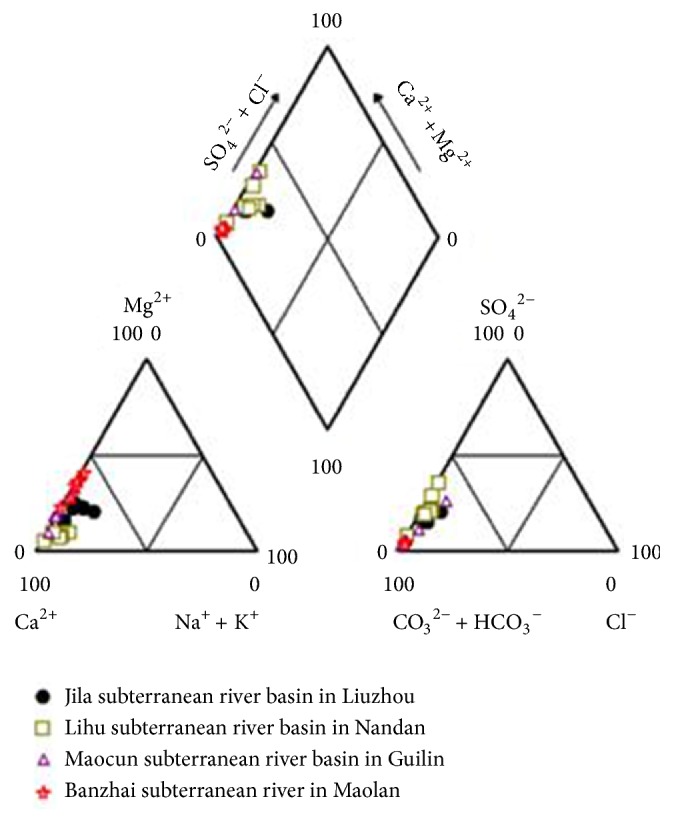
Hydrogeological types in the four subterranean river basins.

The GW_Chart software was used to analyze the hydrochemical types. The results show that the hydrochemical types are either HCO_3_-Ca or HCO_3_-Ca·Mg (Figure 6). Thus, limestone is the main geological component of the subterranean rivers in the four selected study areas.

### 5.3. Correlation Analysis of the Main Hydrochemical Indexes

Because a change in Ec can reflect changes in the degree of groundwater pollution, Ec is an important index in the determination of water quality. The correlation analysis of the measurement results for the water samples collected from the four study areas (Table 6) using SPSS revealed that the main ions K^+^, Na^+^. and SO_4_^2−^ exhibited a significant correlation (p<0.01) and that Ca^2+^, HCO_3_^−^ and Mg^2+^ exhibited a good correlation (p<0.01).

### 5.4. The Development of Ca^2+^, HCO_3_^−^, and Mg^2+^

**Figure 7:**
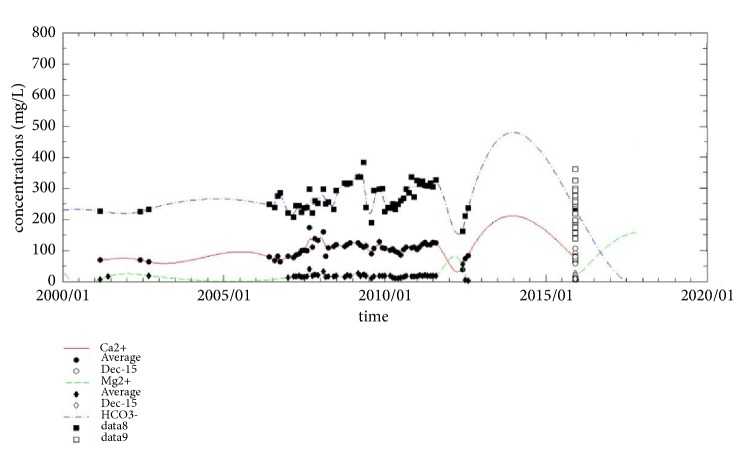
The fitting curves of the means of Ca^2+^, HCO_3_^−^, and Mg^2+^, and the data points of the December 2015 sampling results.

In MATLAB, the cubic spline interpolation method was used to fit all the data. The squared correlation coefficients (R^2^) of the obtained fitting curves for Ca^2+^, HCO_3_^−^, and Mg^2+^ are 0.8545, 0.8689, and 0.7632, respectively. The corrected R^2^ values are 0.6739, 0.7088, and 0.4853, respectively (Figure 7).

Figure 7 shows that the trends of Ca^2+^ and HCO_3_^−^ are very similar. This observation can be attributed to the dissolution of carbonate rock [19], which is mainly due to the greenhouse effect and water acidification. In recent years, the majority of the precipitation in the South China Karst region has mostly been acidic [20]. Furthermore, the greenhouse effect and water acidification can also cause the dissolution of dolomite, releasing Mg ions into solution.

We found from the December 2015 sampling results that, in the study areas heavily affected by human activities, the main ion concentrations in the subterranean rivers are high. In contrast, in the study areas far from urban development and thus less affected by human activities, the main ion concentrations in the subterranean rivers are much lower (shown in Table 1).

### 5.5. The Development of K^+^, Na^+^, Cl^−^, SO_4_^2−^, and NO_3_^−^

In MATLAB, the cubic spline interpolation method was used to fit all the data. The R2 values of the obtained fitting curves for K+ and Na+ are 0.9085 and 0.8964, respectively. The corrected R2 values are 0.7904 and 0.7669, respectively. The R2 values of the obtained fitting curves for Cl-, SO42- and NO3- are 0.7531, 0.6222, and 0.7997, respectively, and the corrected R2 values are 0.5272, 0.2815, and 0.6127, respectively,

Changes in the land use types and land coverage can affect the compositions of infiltrating water [17]. Guo et al. [10] analyzed the changes in the concentrations of major ions in tens of typical subterranean rivers in karst regions in South China over the past 20 years. They found that the K+ and Na+ concentrations exhibited marked increasing trends, and they concluded that the land use type substantially affected the quality of karst ground water. Evaluation of the change patterns in the ions in the Maocun village subterranean river basin over the past 20 years revealed that agricultural activity (e.g., land use, application of fertilizers, domestic waste, domestic sewage, deforestation, and destruction) greatly affected the quality of the ground water. Not only did NO3- and SO42- concentrations increase, but K+, Na+, and Cl- concentrations also increased substantially.

The primary sources for the K+ and Na+ in the water samples collected in December 2015 are pesticides, fertilizers, and domestic sewage, which enter into and reside in the subterranean river systems via different influents and effluents in the karst regions and through soil infiltration.

**Figure 8:**
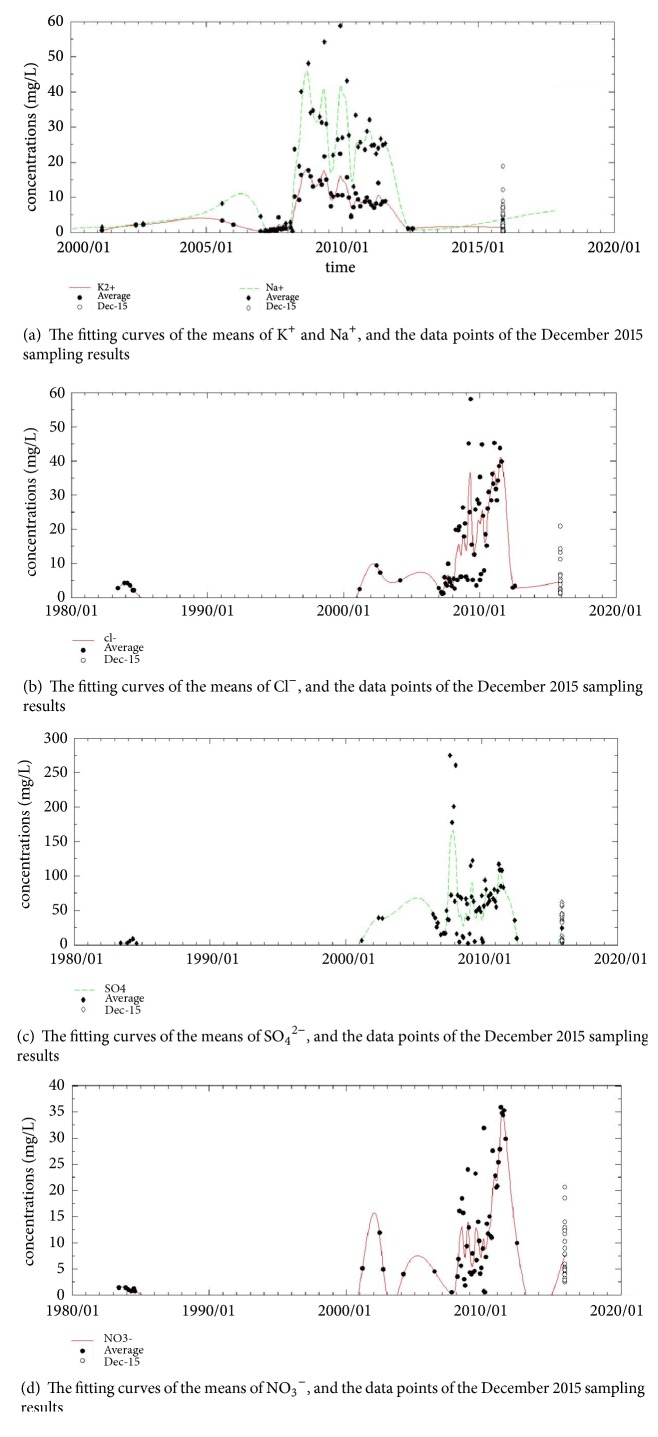


Figures 8(a), 8(b), 8(c), and 8(d) show that the heaviest pollution occurred in approximately 2010. Many research papers have examined this period. The data points obtained from the literature have a wide range, which suggests that development in the South China Karst region is spatially variable. It is believed that the neglect of policy, the rapid development of industrial society, and the development of high-density populations may be the main drivers of changes. Compared to the results from approximately 2010, the results from 2015 demonstrate that the water quality in even the most heavily polluted Jila subterranean river in Liuzhou greatly improved, and this improvement is attributed to the treatment imposed by urban planning and the corresponding policies.

## 6. Conclusions

We consolidated the data collected since 1960 and used the cubic spline interpolation method in MATLAB to fit all the data. Based on the analysis of the main hydrochemical indexes in water samples from subterranean rivers in four study areas in December 2015, the following conclusions can be made:

(1) Development in the South China Karst region is spatially variable. However, although changes in the subterranean rivers occur slowly, the water quality can change rapidly in response to the transformation of industrial society.

(2) The trends of the Ca^2+^ and HCO_3_^−^ concentrations are very similar, and this similarity is mainly attributed to rock weathering from dissolution, which has been enhanced by human activities.

Similarly, the trends of the K^+^ and Na^+^ concentrations are also similar, indicating that domestic sewage, pesticides, and fertilizer are the main sources.

(3) The concentrations of major ions in the subterranean rivers in the 1980s were very low. Since then, the subterranean rivers of the South China Karst region have experienced a dramatic change. In approximately 2010, the pollution in the subterranean rivers in the South China Karst region reached an unprecedented peak, and some the basins of some subterranean rivers have been overdeveloped. It is believed that the neglect of policy, the rapid development of industrial society, and the development of high-density populations may be the main drivers of changes. The transformation of the subterranean rivers in Liuzhou shows the crucial importance of active protection and treatment as well as urban planning that takes into account environmental protection during development in karst regions.

## Figures and Tables

**Table 1:** Data on the main hydrochemical indexes at the 25 sampling sites in the four study areas.

Sampling sites	Indexes											
	pH	T	Ec	DO	K^+^	Na^+^	Ca^2+^	Mg^2+^	HCO_3_^−^	Cl^−^	SO_4_^2−^	NO_3_^−^
		(°C)	(*μ*s/cm)	(mg.L^−1^)	(mg.L^−1^)	(mg.L^−1^)	(mg.L^−1^)	(mg.L^−1^)	(mg.L^−1^)	(mg.L^−1^)	(mg.L^−1^)	(mg.L^−1^)
JL-1	7.35	22.90	507.00	5.81	1.32	5.30	92.00	10.74	268.40	6.49	43.17	9.00
JL-2	7.62	24.00	564.00	7.27	1.60	7.11	90.00	16.40	292.80	11.32	41.30	7.89
JL-3	7.20	23.00	606.00	1.39	1.76	8.95	92.00	19.12	317.20	13.17	44.82	18.60
JL-4	7.05	20.70	544.00	0.01	4.88	12.15	82.00	16.00	256.20	14.24	57.78	14.02
JL-5	7.19	21.40	557.00	1.82	7.05	18.95	78.00	15.37	244.00	20.86	56.51	20.64
ND-1	9.20	25.00	304.00	12.50	2.20	5.66	62.00	3.41	173.85	4.71	34.21	8.95
ND-2	8.19	22.10	375.00	10.54	2.90	7.94	65.00	4.33	170.80	6.82	38.76	12.57
ND-3	7.31	19.40	392.00	5.39	1.11	4.85	76.00	4.24	195.20	3.83	35.77	10.21
ND-4	7.77	20.50	352.00	7.58	2.17	6.59	69.00	3.48	158.60	5.62	34.29	12.91
ND-5	8.05	20.00	350.00	8.97	1.33	2.64	72.00	4.47	140.30	2.60	45.42	6.02
ND-6	8.20	20.30	364.00	8.65	1.90	5.93	70.00	3.31	176.90	4.90	32.82	11.75
ND-7	7.60	20.80	337.00	8.02	0.44	0.98	72.00	2.27	201.30	1.64	12.67	5.32
ND-8	8.09	19.70	352.00	8.22	1.58	1.98	69.00	4.47	140.30	2.41	61.52	5.13
MC-1	6.78	9.30	18.40	11.35	0.20	0.28	19.00	1.10	12.20	1.12	3.66	2.62
MC-2	6.75	12.90	81.30	10.06	0.17	0.27	17.00	3.78	36.60	1.08	3.50	2.70
MC-3	7.62	13.00	155.70	10.61	0.10	0.29	25.00	7.25	103.70	1.09	3.99	2.48
MC-4	7.01	19.30	487.00	8.91	0.32	0.35	106.00	12.42	353.80	1.10	6.10	2.80
MC-5	7.36	17.90	333.00	8.91	0.50	0.48	72.00	9.76	213.50	1.42	5.79	3.93
MC-6	7.93	17.80	355.00	9.32	0.57	0.47	79.00	9.80	250.10	1.43	6.06	4.76
ML-1	7.49	17.80	395.00	7.88	0.15	0.21	72.00	12.83	256.20	1.27	7.55	3.25
ML-2	7.51	13.80	401.00	6.15	1.57	0.69	64.00	15.39	256.20	1.71	7.71	3.90
ML-3	7.66	17.60	427.00	8.10	1.20	0.35	64.00	26.54	286.70	1.58	7.85	4.14
ML-4	7.66	18.10	409.00	7.02	0.73	0.71	62.00	21.09	244.00	1.73	8.69	12.34
ML-5	8.03	15.30	369.00	9.21	0.62	0.39	58.00	18.30	237.90	1.44	7.59	3.97
ML-6	7.85	17.80	386.00	8.79	0.44	0.30	58.00	19.73	250.10	1.30	8.03	4.73

**Table 2:** Comparison of the main indexes in the Jila subterranean river basin (mg.L^−^1).

Sampling sites	Indexes					
	Ca^2+^	NH_4_^+^	SO_4_^2−^	NO_3_^−^	Mn	Hg
JL-1 (2015)	92.00	0.00	43.17	9.00	<0.06*∗*10^−3^	0
JL-2 (2015)	90.00	0.04	41.30	7.89	<0.06*∗*10^−3^	0
JL-3 (2015)	92.00	0.00	44.82	18.60	<0.06*∗*10^−3^	0
JL-4 (2015)	82.00	1.94	57.78	14.02	<0.06*∗*10^−3^	0
JL-5 (2015)	78.00	3.36	56.51	20.64	<0.06*∗*10^−3^	0
Average of JL-1~ JL-5	86.80	1.07	48.72	14.03	<0.06*∗*10^−3^	0
Max of Reference [14]		3.14	81.34	18.19	0.28	
Max of Reference [15]	99.16		76.95		0.52	0.01

**Table 3:** Comparison of the main indexes in the Lihu subterranean river basin (mg.L^−^1).

Sampling sites	Indexes								
	K^+^	Na^+^	Ca^2+^	Mg^2+^	HCO_3_^−^	Cl^−^	SO_4_^2−^	Mn	As
ND-1 (2015)	2.20	5.66	62.00	3.41	173.85	4.71	34.21	0.072*∗*10^−3^	11.3*∗*10^−3^
ND-2 (2015)	2.90	7.94	65.00	4.33	170.80	6.82	38.76	0.4*∗*10^−3^	11.3*∗*10^−3^
ND-3 (2015)	1.11	4.85	76.00	4.24	195.20	3.83	35.77	<0.06*∗*10^−3^	1.3*∗*10^−3^
ND-4 (2015)	2.17	6.59	69.00	3.48	158.60	5.62	34.29	<0.06*∗*10^−3^	11.4*∗*10^−3^
ND-5 (2015)	1.33	2.64	72.00	4.47	140.30	2.60	45.42	0.17*∗*10^−3^	3.62*∗*10^−3^
ND-6 (2015)	1.90	5.93	70.00	3.31	176.90	4.90	32.82	0.1*∗*10^−3^	11*∗*10^−3^
ND-7 (2015)	0.44	0.98	72.00	2.27	201.30	1.64	12.67	<0.06*∗*10^−3^	<0.09*∗*10^−3^
ND-8 (2015)	1.58	1.98	69.00	4.47	140.30	2.41	61.52	0.97*∗*10^−3^	4.93*∗*10^−3^
Average of ND-1~ ND-8	1.70	4.57	69.38	3.75	169.66	4.07	36.93		
Max of Reference [6]	2.64	1.74	65.68	8.82	236.42	6.47	60.78	0.59	0.04

**Table 4:** Comparison of the main indexes in the Maocun subterranean river basin (mg.L^−^1).

Sampling sites	Indexes		
	Ca^2+^	Mg^2+^	HCO_3_^−^
MC-1 (2015)	19.00	1.10	12.20
MC-2 (2015)	17.00	3.78	36.60
MC-3 (2015)	25.00	7.25	103.70
MC-4 (2015)	106.00	12.42	353.80
MC-5 (2015)	72.00	9.76	213.50
MC-6 (2015)	79.00	9.80	250.10
Average MC-4~ MC-6	85.67	10.66	279.17
Max of Reference [9]	94.30	11.51	298.20

**Table 5:** Comparison of the main indexes in the Banzhai subterranean river basin (mg.L^−^1).

Sampling sites	Indexes					
	K^+^	Na^+^	Ca^2+^	Mg^2+^	HCO_3_^−^	SO_4_^2−^
ML-1 (2015)	0.15	0.21	72.00	12.83	256.20	7.55
ML-2 (2015)	1.57	0.69	64.00	15.39	256.20	7.71
ML-3 (2015)	1.20	0.35	64.00	26.54	286.70	7.85
ML-4 (2015)	0.73	0.71	62.00	21.09	244.00	8.69
ML-5 (2015)	0.62	0.39	58.00	18.30	237.90	7.59
ML-6 (2015)	0.44	0.30	58.00	19.73	250.10	8.03
Average of ML-1~ ML-6	0.79	0.44	63.00	18.98	261.46	7.90
Max of Reference [16]	1.60	1.80	86.00	57.19	274.50	13.64

**Table 6:** Correlation analysis of the main hydrochemical indexes.

	Ec	K^+^	Na^+^	Ca^2+^	Mg^2+^	Cl^−^	SO_4_^2−^	HCO_3_^−^	NO_3_^−^
Ec	1								
*K* ^*+*^	0.482^*∗*^	1							
Na^+^	0.579^*∗∗*^	0.931^*∗∗*^	1						
Ca^2+^	0.787^*∗∗*^	0.190	0.316	1					
Mg^2+^	0.516^*∗*^	0.035	-0.029	0.09	1				
Cl^−^	0.706^*∗∗*^	0.875^*∗∗*^	0.964^*∗∗*^	0.385	0.154	1			
SO_4_^2−^	0.453^*∗*^	0.702^*∗∗*^	0.771^*∗∗*^	0.349	-0.264	0.724^*∗∗*^	1		
HCO_3_^−^	0.761^*∗∗*^	-0.004	0.043	0.659^*∗∗*^	0.731^*∗∗*^	0.217	-0.196	1	
NO_3_^−^	0.559^*∗∗*^	0.769^*∗∗*^	0.881^*∗∗*^	0.283	0.034	0.844^*∗∗*^	0.669^*∗∗*^	0.055	1

*Note.* ^*∗*^ indicates a significant correlation at the 0.05 level, ^*∗∗*^ indicates a significant correlation at the 0.01 level (bilateral), and h=22.

## Data Availability

All data were provided by the Program for Geological Survey Projects in Institute of karst Geology of China (2015003), and also some data cannot be allowed to present in the manuscript.
